# Osteopontin Deficiency Accelerates Spontaneous Colitis in Mice with Disrupted Gut Microbiota and Macrophage Phagocytic Activity

**DOI:** 10.1371/journal.pone.0135552

**Published:** 2015-08-14

**Authors:** Takahiko Toyonaga, Hiroshi Nakase, Satoru Ueno, Minoru Matsuura, Takuya Yoshino, Yusuke Honzawa, Ayako Itou, Kazuyoshi Namba, Naoki Minami, Satoshi Yamada, Yorimitsu Koshikawa, Toshimitsu Uede, Tsutomu Chiba, Kazuichi Okazaki

**Affiliations:** 1 Department of Gastroenterology and Hepatology, Kansai Medical University, Hirakata, Osaka, Japan; 2 Department of Gastroenterology and Hepatology, Graduate School of Medicine, Kyoto University, Sakyo-ku, Kyoto, Japan; 3 Department of Internal medicine, Takashima Municipal Hospital, Takashima, Shiga, Japan; 4 Nutritional Science Institute, Morinaga Milk Industry Co., Ltd, Zama, Kanagawa, Japan; 5 Department of Matrix Medicine, Institute for Genetic Medicine, Hokkaido University, Sapporo, Hokkaido, Japan; Laikon Hospital, GREECE

## Abstract

**Background:**

Osteopontin (OPN) is a multifunctional protein expressed in a variety of tissues and cells. Recent studies revealed increased OPN expression in the inflamed intestinal tissues of patients with inflammatory bowel disease (IBD). The role of OPN in the pathophysiology of IBD, however, remains unclear.

**Aims:**

To investigate the role of OPN in the development of intestinal inflammation using a murine model of IBD, interleukin-10 knock out (IL-10 KO) mice.

**Methods:**

We compared the development of colitis between IL-10 KO and OPN/IL-10 double KO (DKO) mice. OPN expression in the colonic tissues of IL-10 KO mice was examined by fluorescence in situ hybridization (FISH) analysis. Enteric microbiota were compared between IL-10 KO and OPN/IL-10 DKO mice by terminal restriction fragment length polymorphism analysis. The effect of OPN on macrophage phagocytic function was evaluated by phagocytosis assay.

**Results:**

OPN/IL-10 DKO mice had an accelerated onset of colitis compared to IL-10 KO mice. FISH analysis revealed enhanced OPN synthesis in the colonic epithelial cells of IL-10 KO mice. OPN/IL-10 DKO mice had a distinctly different enteric bacterial profile with a significantly lower abundance of *Clostridium* subcluster XIVa and a greater abundance of *Clostridium* cluster XVIII compared to IL-10 KO mice. Intracellular OPN deletion in macrophages impaired phagocytosis of fluorescence particle-conjugated *Escherichia coli in vitro*. Exogenous OPN enhanced phagocytosis by OPN-deleted macrophages when administered at doses of 1 to 100 ng/ml, but not 1000 ng/ml.

**Conclusions:**

OPN deficiency accelerated the spontaneous development of colitis in mice with disrupted gut microbiota and macrophage phagocytic activity.

## Introduction

Osteopontin (OPN), a phosphorylated glycoprotein originally identified in bone, binds to hydroxyapatite and regulates ectopic calcium deposition [1]. OPN is also expressed in various other tissues and cells, including intestinal epithelial cells and immune cells, such as macrophages, dendritic cells, and T lymphocytes [2]. Two forms of OPN with distinct functions have been identified [3]. One is the classical form of OPN secreted from various cells, including intestinal epithelial cells and activated T lymphocytes (secreted form of OPN [sOPN]), and the other is the intracellular form of OPN (iOPN), which was identified in macrophages [4] and dendritic cells [5]. Although macrophages constitutively express iOPN, they hardly secrete any OPN, even after stimulation with lipopolysaccharides (LPS) [6].

OPN is implicated in macrophage migration and several macrophage functions [3]. Generally, exogenous OPN enhances macrophage migration *in vitro* and *in vivo* [7]. Exogenous OPN works as a pro-inflammatory cytokine by inducing interleukin-12 (IL-12) and suppressing IL-10 expression in macrophages through interactions with αvβ3 integrin and CD44 [8]. Previous studies in OPN-deficient mice revealed that OPN is essential for efficient development of T-helper 1 immune responses, and thus plays an important role in protecting against microbial and viral infection [9, 10]. OPN deficiency in macrophages can also result in impaired phagocytosis [11]. Thus, OPN has a crucial role in the development of both adaptive and innate immune responses.

Inflammatory bowel disease (IBD) is a relapse-remitting disorder characterized by recurrent intestinal inflammation throughout the gastrointestinal tract. The precise pathophysiology of IBD remains unknown, but a dysregulated immune response toward enteric bacteria is a strong component [12]. Especially, intestinal macrophages underlying the subepithelial lamina propria play a crucial role in not only intestinal homeostasis but also in the pathophysiology of IBD by responding to commensal microbes. Indeed, biologic agents targeting tumor necrosis factor-alpha (TNF-α), a representative cytokine secreted from activated macrophages, are well-known for their efficacy in the clinical treatment of IBD [13]. Increased levels of OPN in the intestinal mucosa and the serum of patients with active IBD suggest that OPN is involved in the development of intestinal inflammation [14–16]. Experimental studies regarding the effects of OPN on colonic inflammation, however, have produced conflicting results. In the acute phase of dextran sulfate sodium (DSS)-induced colitis, one study reported exacerbated colitis in OPN-deficient mice compared to wild-type mice with reduced nitric oxide synthase (NOS) expression and impaired phagocytic activity in intestinal macrophages, whereas another study reported attenuated colitis with reduced macrophage migration in the inflamed colonic tissues of OPN-deficient mice [11, 17]. On the other hand, attenuated colitis was reported in the chronic phase of DSS- and trinitrobenzene sulfonic acid-induced colitis [11, 18]. Thus, whether OPN ameliorates or exacerbates colonic inflammation is unclear, despite emerging evidence that OPN is associated with the pathophysiology of human IBD [11, 17–19].

In the present study, we investigated the role of OPN in the development of colitis using a murine experimental colitis model: interleukin-10 knockout (IL-10 KO) mice that develop spontaneous colitis in response to enteric bacteria [20]. Like human IBD, dysregulated activation of intestinal macrophages plays a crucial role in the development of colitis in IL-10 KO mice, as demonstrated in mice lacking IL-10 receptors or signal transducers and activators of transcription 3 in a macrophage-specific manner [21, 22]. Our findings revealed that OPN deficiency accelerated the development of spontaneous colitis in mice with disrupted gut microbiota and macrophage phagocytic activity.

## Materials and Methods

### Ethics statement

This study was performed in strict accordance with the recommendations in the Guide for the Care and Use of Laboratory Animals of the National Institutes of Health. The protocol was approved by the Animal Protection Committee of Kyoto University (Approval Number MedKyo14291). All mice were euthanized by cervical dislocation under diethyl ether anesthesia, and all efforts were made to minimize suffering.

### Mice

The C57BL/6NCrSlc wild-type (WT) mice were purchased from Japan SLC (Shizuoka, Japan). The B6.129P2-*Il10* ^*tm1Cgn*^/J (IL-10 KO) mice were purchased from Charles River Japan (Kanagawa, Japan). The OPN KO mice were a kind gift from Toshimitsu Uede (Department of Matrix Medicine, Institute for Genetic Medicine, Hokkaido University). The OPN/IL-10 DKO mice were generated by intercrossing OPN KO and IL-10 KO mice. The mice were fed standard laboratory chow and supplied drinking water *ad libitum*. All mice were confirmed negative for Sendai virus, mouse hepatitis virus, ectomelia virus, LCM virus, *Mycoplasma spp*., *Clostridium piliforme*, *Corynebacterium kutscheri*, and *Salmonella spp*. based on monitoring during the experiments. We used 48 WT, 74 IL-10 KO, 24 OPN KO, and 74 OPN/IL-10 DKO female mice at 3 to 12 weeks of age for the experiments.

### Clinical score

Clinical signs of IL-10 KO and OPN/IL-10 DKO mice were evaluated over time, as previously described [23]. Each of the following subscores was given a value of either 0 or 1: excreted perianal mucus, rectal prolapse, diarrhea, or weight loss greater than 5%. The total clinical score was the sum of the four subscores.

### Microscopic assessment of colitis

The mice were euthanized, and the entire colon was removed. Rectums were dissected, fixed in 10% formaldehyde, dehydrated in ethanol, and embedded in paraffin. Transverse sections were prepared, stained with hematoxylin and eosin (HE), and histologically analyzed in a blind manner. Histologic damage was quantified by the histologic scoring systems as described previously [24].

### Quantitative analysis of gene expression

Collected rectal tissues were quickly frozen in liquid nitrogen for later extraction of the mRNA. Total RNA was extracted using the guanidium isothiocyanate-phenol-chloroform method. Extracted RNA was reverse-transcribed with SuperScript II Reverse Transcriptase (Invitrogen, Carlsbad, CA, USA) and the resulting complementary DNAs were analyzed for the expression of *glyceraldehyde phosphate dehydrogenase* (*GAPDH*), *interleukin-6* (*IL-6*), *interferon-γ* (*IFN-γ*), *interleukin-17A* (*IL-17A*), and *CD11b* mRNA using the LightCycler 480 System II (Roche Applied Science, Indianapolis, IN, USA). The primer sequences were as follows: *GAPDH*, 5’-CAA CTT TGT CAA GCT CAT TTC C-3’ (forward), 5’-GGT CCA GGG TTT CTT ACT CC-3’ (reverse); *IL-6*, 5’-AGT CCG GAG AGG AGA CTT CA-3’ (forward), 5’-ATT TCC ACG ATT TCC CAG AG-3’ (reverse); *IFN-γ*; 5’-AGC TCT TCC TCA TGG CTG TT-3’ (forward), 5’-ATC TGG CTC TGC AGG ATT TT-3’ (reverse); *IL-17A*, 5’-TCT CTG ATG CTG TTG CTG CT-3’ (forward), 5’-CGT GGA ACG GTT GAG GTA GT-3’ (reverse); and *CD11b*, 5’-GCT CCG GTA GCA TCA ACA AC-3’ (forward), 5’-AGT GAA TCC GGA ACT CGT CCG-3’ (reverse). The resulting gene expression levels of target molecules were normalized based on the expression of *GAPDH*.

### Fluorescence in situ hybridization

For detection of the *secreted phosphoprotein 1* gene coding OPN, we adapted Quantigene ViewRNA (Affymetrix, Santa Clara, CA, USA) using a custom probe set targeting the *secreted phosphoprotein 1* gene (Affymetrix; GenBank Accession Number. NM_009263). Fluorescence *in situ* hybridization (FISH) was performed according to the manufacturer’s instructions. Briefly, rectal tissues collected from 4-week-old WT, IL-10 KO, and OPN/IL-10 DKO mice were quickly frozen in OCT and dissected to prepare 5-μm transverse sections. After fixation, washing, and dehydration in ethanol, the sections were digested in Protease GF (Affymetrix) and then hybridized with custom-designed QuantiGene ViewRNA probes against target RNA sequences. Bound probes were preamplified and subsequently amplified according to the manufacturer’s instructions. Probe oligonucleotide conjugated to alkaline phosphatase type 1 was added, following by Fast Red substrate. Nuclei were visualized by Hoechst staining. In some experiments, the sections were further incubated with rabbit anti-mouse E-cadherin antibody (1:100 dilution; Abcam, Cambridge, MA, USA), and signals were visualized by Alexa Fluor 488-conjugated anti-rabbit antibody (1:1000 dilution; Invitrogen). Images were acquired using a fluorescence microscope (BIOREVO BZ-9000; Keyence, Osaka, Japan) with the BZ-Analyzer version 2.1 software and composed in Photoshop Elements 12 (Adobe Systems Inc., San Jose, CA, USA).

### Preparation and stimulation of bone marrow-derived macrophages

Murine bone marrow cells were obtained from femur and tibia bone marrow. Cells were cultured in complete Roswell Park Memorial Institute (RPMI) 1640 medium (Gibco, Invitrogen, Grand Island, NY, USA) containing 10% heat-inactivated fetal bovine serum, 100 mg/ml of streptomycin (Sigma-Aldrich, St. Louis, MO, USA), 100 mg/ml of penicillin (Sigma-Aldrich), and 40 ng/ml of recombinant macrophage colony-stimulating factor (Peprotech, Rocky Hill, NJ, USA) for 5 d at 37°C. Bone marrow-derived macrophages (BMDMs) were harvested and seeded at 5×10^5^ cells/well in 6-well culture plates (Greiner Bio-One, Monroe, NC, USA). After overnight incubation with 10 ng/ml IFN-*γ*(R&D Systems, Minneapolis, MN, USA), the wells were washed and challenged with stimulators as follows: Pam3CSK4 (Toll-like receptor [TLR]2 ligand) 50 to 500 ng/ml, LPS (TLR4 ligand) 100 ng/ml, and ODN1585 (TLR9 ligand) 500 nM (Pam3CSK4 and ODN1585 from InvivoGen [San Diego, CA, USA] and LPS from Sigma-Aldrich). The cells were stimulated for 24 h before the supernatants were collected and stored at -80°C until assayed.

### Enzyme-linked immunosorbent assay

The TNF-α, IL-6, and interleukin-12p40 (IL-12p40) levels in the cell supernatants were quantified using a mouse enzyme-linked immunosorbent assay (ELISA) kit (eBioscience, San Diego, CA, USA) according to the manufacturer’s instructions.

### Isolation of lamina propria mononuclear cells

Lamina propria mononuclear cells (LPMCs) were isolated from colonic tissues of IL-10 KO and OPN/IL-10 DKO mice as previously described [25]. Briefly, the entire colons were removed from euthanized mice and washed with phosphate-buffered saline (PBS). After disrupting epithelial cells in Hank’s Balanced Salt Solution containing 5 mM EDTA and 1 mM dithiothreitol, the colonic tissues were repeatedly digested in PBS containing 0.5 mg/ml collagenase D (Roche Applied Science), 0.5 mg/ml DNase I (Sigma-Aldrich), and 3 mg/ml Dispase II (Roche Applied Science) at 37°C for 30 minutes under slow rotation. Collected supernatants were spun down, and the obtained pellet was re-suspended in 40% Percoll solution and overlaid on 80% Percoll solution. LPMCs were obtained from the interphase and washed with PBS. Total RNA was extracted from LPMCs, and gene expression was analyzed as described above. The primer sequences were as follows: *nitric oxide synthase 2* (*NOS2*), 5’-ATG CAA CAT GGG AGC CAC AGC-3’ (forward), 5’-CTG GGA TTT CAG CCT CAT GG-3’ (reverse); *TNF-α*, 5’-CAT GCA CCA CCA TCA AGG AC-3’ (forward), 5’-GGC CTG AGA TCT TAT CCA GCC-3’ (reverse); *IL-12p40*, 5’-GGA AGC ACG GCA GCA GAA TA-3’ (forward), 5’-TGA CCT CCA CCT GTG AGT TC-3’ (reverse).

### Terminal restriction fragment length polymorphism analysis

Terminal restriction fragment length polymorphism (T-RFLP) analysis was performed as described previously [26]. Briefly, murine cecal contents were suspended in a solution containing 100 mM Tris-HCl (pH 9.0), 40 mM EDTA, 4 M guanidine thiocyanate, and 0.001% bromothymol blue. Fecal solids in the suspension were broken down using the FastPrep 24 Instrument (MP Biomedicals, Irvine, CA, USA) with zirconia beads. Fecal DNA was extracted from the suspension by an automatic nucleic acid extractor (Precision System Science, Chiba, Japan) using MagDEA DNA 200 (GC) (Precision System Science) as the reagent.

The 16S rRNA gene was amplified from fecal DNA using the primers of 5’-FAM-labeled 516f (5’-TGCCAGCAGCCGCGGTA-3’; *Escherichia coli* [*E*. *coli*] positions 516 to 532) and 1510r (5’-GGTTACCTTGTTACGACTT-3’; *E*. *coli* positions 1510 to 1492). The resulting 16S rDNA amplicons were treated with 10 U of *Bsl* l (New England BioLabs, MA, USA) for 3 hours, and the digestives were fractionated using an ABI PRISM 3130xl Genetic Analyzer (Applied Biosystems, Foster City, CA, USA) with the DNA analysis software Gene Mapper. The major terminal restriction fragments similar in size of 1–3 bp were summarized to operational taxonomic units (OTUs), and the corresponding bacterial groups were estimated by computer simulation based on the human intestinal microbiota database [27]. The percentage of each bacterial group to total enteric bacteria was represented by the percentage of each summed OTU area to total OTU area [28]. Cluster analyses were performed using Gene Maths software (Applied Maths, Kortrijk, Belgium) based on the obtained T-RFLP profiles, and the similarity among samples was evaluated by principal component analysis.

### Preparation of thioglycollate-elicited peritoneal macrophages

Mice were intraperitoneally injected with 3 ml of 4% thioglycollate (Eilken Chemical Co., Tokyo, Japan) and peritoneal exudate cells were harvested on Day 4. After removing erythrocytes, the cells were subjected to positive selection using anti-CD11b magnetic microbeads (Miltenyi Biotec, Auburn, CA, USA) to select CD11b-positive thioglycollate-elicited peritoneal macrophages (TEPMs).

### Phagocytosis assay

A phagocytosis assay was performed using pHrodo Green *E*. *coli* BioParticles Conjugate (Invitrogen) according to the manufacturer’s instructions. Briefly, TEPMs were seeded at 2.5×10^5^ cells/well in 96-well cell culture plates (Greiner Bio-One) and incubated overnight in complete RPMI 1640 medium containing 40 ng/ml recombinant macrophage colony-stimulating factor. The wells were washed with PBS and challenged with fluorescent particles diluted in Live Cell Imaging Solution (Invitrogen). In several wells, recombinant OPN (rOPN, R&D Systems) was added at a concentration of 1, 10, 100, or 1000 ng/ml. The cells were incubated at 37°C and fluorescence intensity was measured at the indicated times using a fluorescence microplate reader (Infinite F200 PRO; TECAN, Kawasaki, Japan). Phagocytic activity was evaluated by subtracting the average fluorescence intensity of the no-cell negative-control wells from that of all experimental wells and indicated as the relative value.

### Identification of bacterial species in the mesenteric lymph node

Bacterial species in the mesenteric lymph nodes (MLNs) of the mice were detected as previously reported with some modifications [29]. MLNs were obtained from mice under sterile conditions and mechanically disrupted with zirconia beads. Bacterial DNA was extracted using a bacterial DNA/RNA extraction kit (MORA-EXTRACT; Kyokuto Pharmaceuticals, Tokyo, Japan). After the first amplification of bacterial 16S rRNA with universal primers (27F; 5’-AGA GTT TGA TCC TGG CTC AG-3’, 1492R; 5’-GGT TAC CTT GTT ACG ACT T-3’), nested PCR was performed using internal primers (35F; 5’-CCT GGC TCA GGA TGA ACG-3’, 519R; 5’-ATT ACC GCG GCK GCT C-3’). The amplified product was electrophoresed on a 1% agarose gel, and purified from the agarose gel using the QIAEX II Gel Extraction Kit (QIAGEN, Düsseldorf, Germany). The purified product was ligated into the pCR4-TOPO vector, and transformed into *E*. *coli* TOP10 cells using the TOPO-TA Cloning Kit (Invitrogen).

The 16S rRNA genes were amplified from cultures using the TempliPhi system (GE Healthcare, NJ, US), and these were used as templates for DNA sequencing. The products were analyzed using an ABI 3730 DNA Analyzer (Applied Biosystems). All nucleotide sequences were checked by BLAST analysis against all entries in the DNA Data Bank of Japan database and aligned using DNASIS Pro (Hitachi Software Engineering, Yokohama, Japan).

### Statistical analysis

All numerical data are expressed as means ± SD or SEM. Differences between groups were analyzed using a non-parametric Mann-Whitney U test or one-way analysis of variance with Bonferroni’s correction for multiple comparisons. A *P* value of less than 0.05 was considered significant.

## Results

### Disruption of OPN accelerated the onset of colitis in IL-10 KO mice

**Fig 1:**
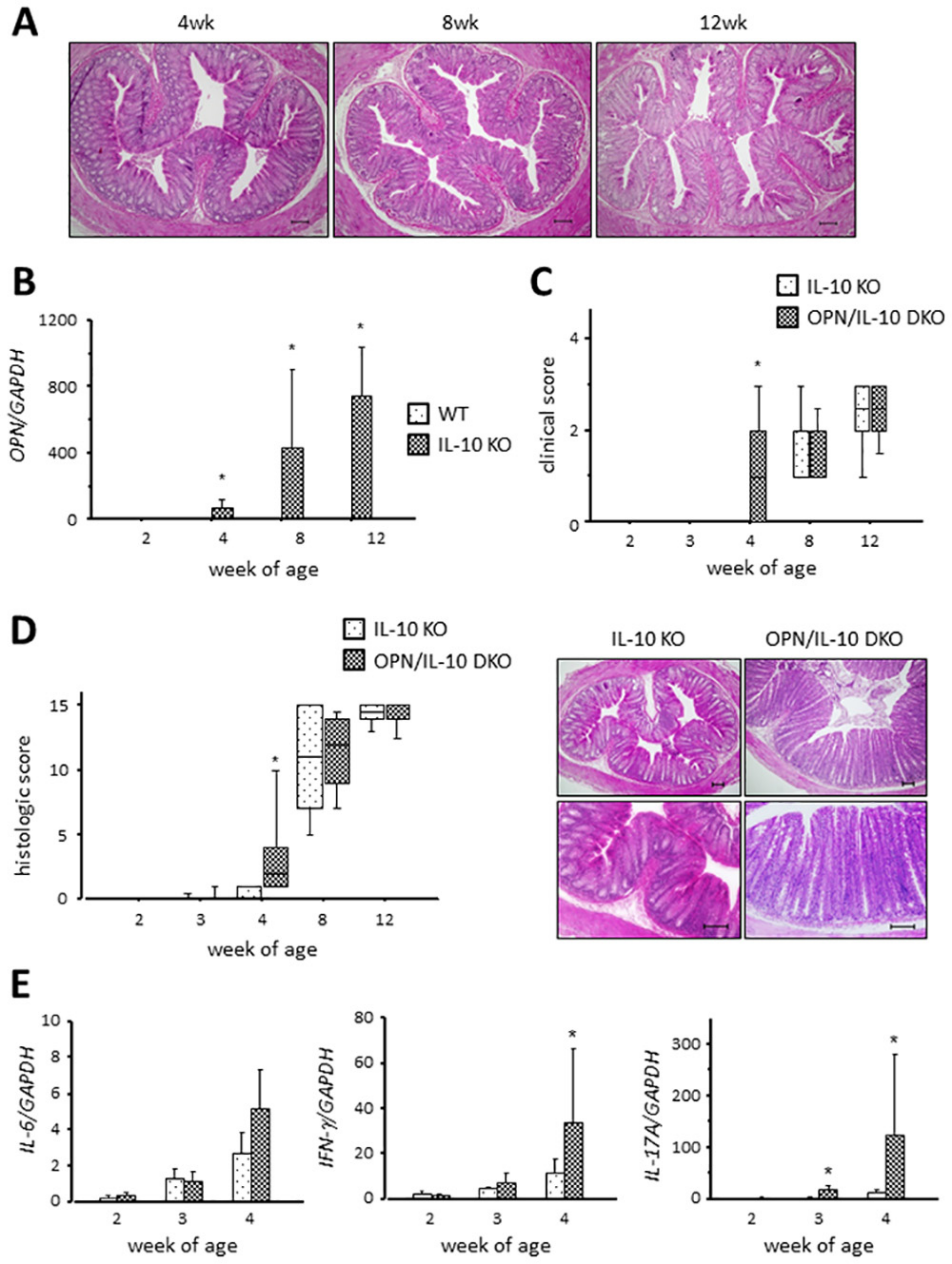
The accelerated onset of colitis in OPN/IL-10 DKO mice compared to IL-10 KO mice. (**A**) Representative HE-staining images of rectal tissues from OPN KO mice at 4, 8, and 12 weeks of age. Scale bar = 100 μm. wk, week of age. (**B**) Gene expression of *OPN* in rectal tissues of WT and IL-10 KO mice. N = 6 for each group. Gene expression of *OPN* was normalized by *GAPDH*. (**C**) Time-dependent changes in clinical score of IL-10 KO and OPN/IL-10 DKO mice. N = 10 for each group. (**D**) Time-dependent changes in histologic scores (left) and representative HE-staining images of rectal tissues from IL-10 KO and OPN/IL-10 DKO mice at 4 weeks of age (right). N = 10 for each group. Scale bar = 100 μm. (**E**) Gene expression of *IL-6*, *IFN-γ*, and *IL-17A* in rectal tissues from IL-10 KO and OPN/IL-10 DKO mice at 4 weeks of age. N = 6 for each group. Gene expression of each target molecule was normalized by *GAPDH*. Error bars represent SD. *P<0.05 compared with WT (B) and IL-10 KO (C-E) mice by non-parametric Mann-Whitney U test.

OPN KO mice did not develop spontaneous colitis (Fig 1A). Therefore, we first deleted OPN in IL-10 KO mice that had increased *OPN* mRNA expression in the colon from 4 weeks of age (Fig 1B), and compared the development of colitis in these mice with that in IL-10 KO mice. Clinical signs, such as perianal mucus excretion and diarrhea, appeared in OPN/IL-10 DKO mice from 4 weeks of age, whereas IL-10 KO mice showed no sign at this time (Fig 1C). Histochemical examination revealed the initiation of colitis between 3 and 4 weeks of age in both IL-10 KO and OPN/IL-10 DKO mice. OPN/IL-10 DKO mice rapidly developed colitis within 1 week, however, and had increased infiltrating immune cells and remarkable epithelial hyperplasia in the rectum compared to IL-10 KO mice at 4 weeks of age (Fig 1D). The histologic score was significantly higher in OPN/IL-10 DKO mice than in IL-10 KO mice at 4 weeks of age, but there was no significant difference in the histologic score at 8 and 12 weeks of age between IL-10 KO and OPN/IL-10 DKO mice. Gene expression of several pro-inflammatory cytokines, such as *IL-6*, *IFN-γ*, and *IL-17A* was upregulated in the rectal tissues of 4-week-old OPN/IL-10 DKO mice compared with IL-10 KO mice (Fig 1E).

### OPN synthesis was enhanced in the colonic epithelial cells of IL-10 KO mice

**Fig 2:**
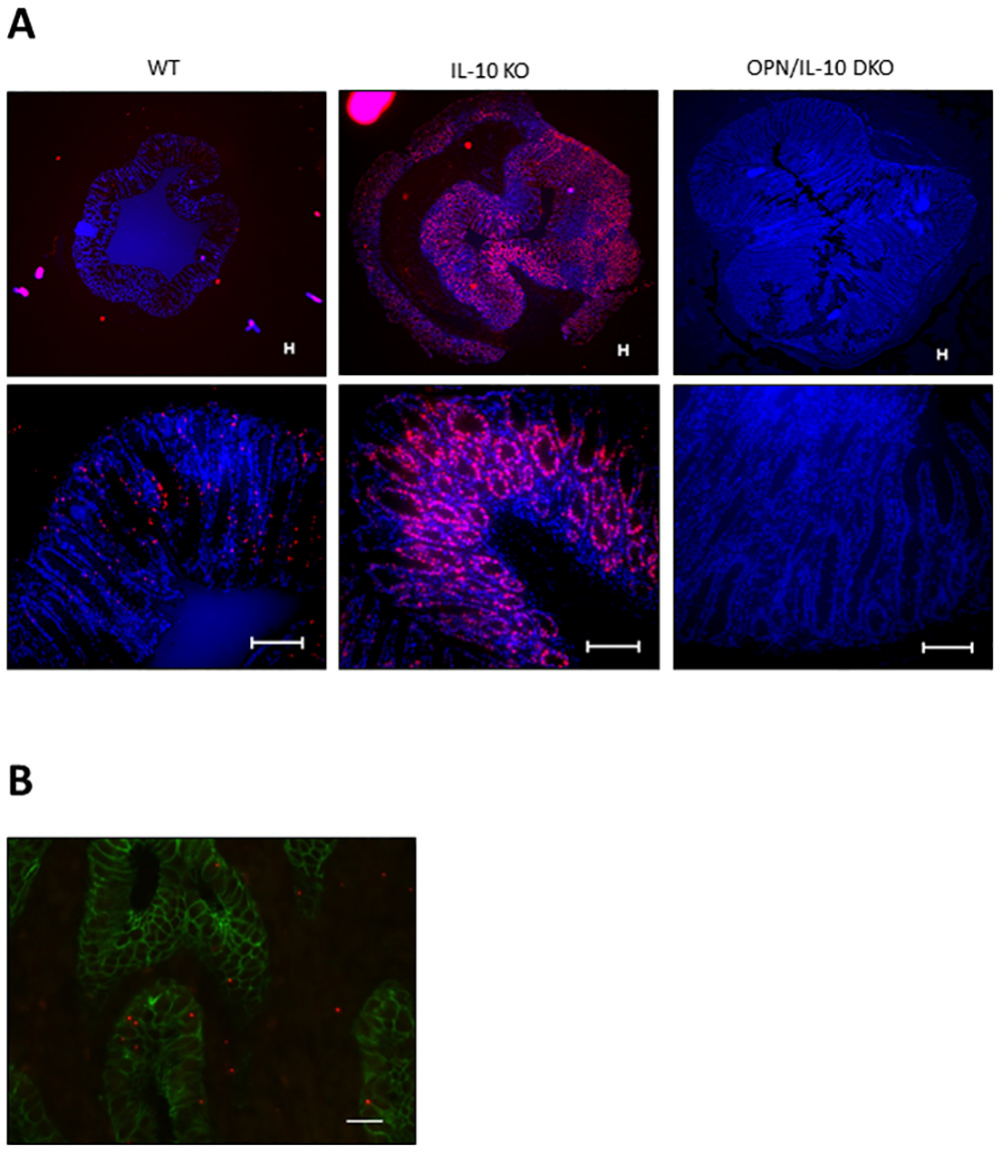
(**A**) Enhanced *OPN* mRNA synthesis in the colonic tissues of IL-10 KO mice. Representative images of FISH for *OPN* mRNA in the rectal tissues from WT, IL-10 KO, and OPN/IL-10 DKO mice at 4 weeks of age. Scale bar = 100 μm. (**B**) Enhanced *OPN* expression localized to the colonic epithelial cells. Representative images of FISH for *OPN* mRNA (red) and immunofluorescent staining for E-cadherin (green) in the rectal tissues from IL-10 KO mice at 4 weeks of age. Scale bar = 100 μm.

We next performed FISH analysis of OPN to determine the main cell type that synthesizes OPN in the colonic tissues of IL-10 KO mice. The FISH analysis revealed remarkably enhanced *OPN* mRNA synthesis in the rectal tissues of 4-week-old IL-10 KO mice compared with WT mice (Fig 2A). OPN synthesis was observed in the E-cadherin expressing colonic epithelial cells of IL-10 KO mice (Fig 2B).

### OPN deficiency affected enteric microbiota in IL-10 KO mice

**Fig 3:**
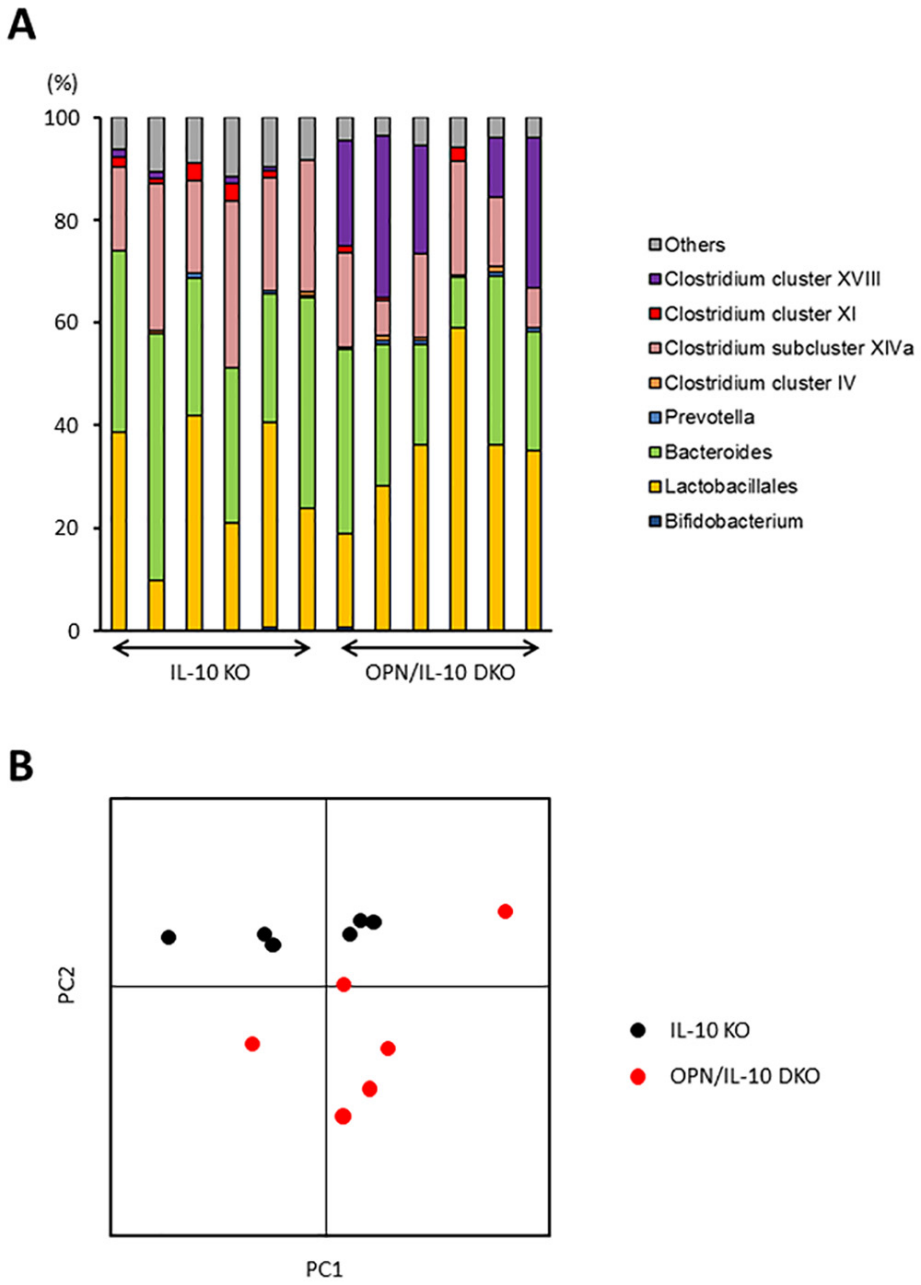
Difference in enteric bacterial composition between IL-10 KO and OPN/IL-10 DKO mice. (**A**) T-RFLP profiles of fecal samples from IL-10 KO and OPN/IL-10 DKO mice at 3 weeks of age. N = 6 for each group. (**B**) Principal component analysis of the T-RFLP profiles. N = 6 for each group. PC, principal component.

To elucidate the role of OPN secreted from colonic epithelia in the innate immune responses, we examined the impact of OPN deficiency on enteric bacteria in IL-10 KO mice. We compared the enteric microbiota between IL-10 KO and OPN/IL-10 DKO mice at 3 weeks of age by T-RFLP analysis. The T-RFLP profiles showed significantly lower abundance of *Clostridium* subcluster XIVa and greater abundance of *Clostridium* cluster XVIII in OPN/IL-10 DKO mice compared to IL-10 KO mice (Fig 3A, Table 1). Principal component analysis of the T-RFLP profiles revealed a distinct difference in the enteric bacterial composition between IL-10 KO and OPN/IL-10 DKO mice (Fig 3B).

**Table 1:** T-RFLP profiles of fecal samples from IL-10 KO and OPN/IL-10 DKO mice.

Predicted bacteria	IL-10 KO	OPN/IL-10 DKO	*P* values
Bifidobacterium	0.1 ± 0.2	0.1 ± 0.2	0.9020
Lactobacillales	29.1 ± 12.9	35.5 ± 13.4	0.8728
Bacteroides	34.4 ± 8.8	24.8 ± 9.5	0.1495
Prevotella	0.3 ± 0.4	0.5 ± 0.3	0.2521
Clostridium cluster IV	0.2 ± 0.4	0.5 ± 0.4	0.3032
Clostridium subcluster XIVa	23.9 ± 6.2	14.2 ± 6.0	0.0374*
Clostridium cluster XI	1.8 ± 1.3	0.8 ± 1.1	0.1659
Clostridium cluster XVIII	0.9 ± 0.7	19.0 ± 11.7	0.0347*
Others	9.3 ± 1.8	4.5 ± 0.9	0.0039*

Each value indicates the percentage of individual predicted bacteria to the total enteric bacteria. N = 6 for each group.

Values are expressed as mean ± SD.

*P<0.05 by non-parametric Mann-Whitney U test.

### Both sOPN and iOPN affected the phagocytic function of macrophages

**Fig 4:**
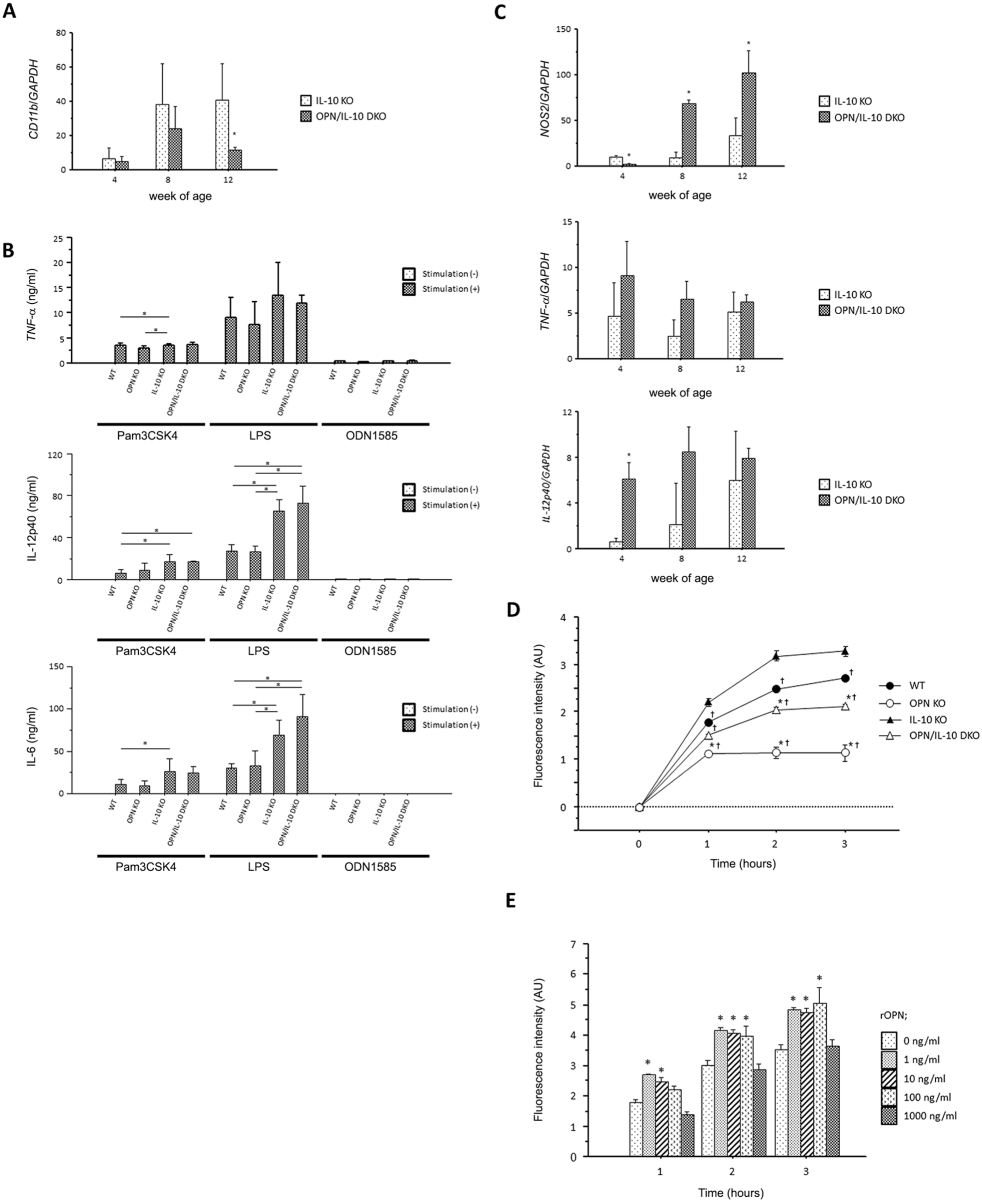
The impact of OPN on cytokine production and phagocytic function of macrophages. (**A**) Gene expression of *CD11b* in rectal tissues from IL-10 KO and OPN/IL-10 DKO mice at 4, 8, and 12 weeks of age. N = 5 for each group. Gene expression of *CD11b* was normalized by *GAPDH*. Error bars represent SD. *P<0.05 compared with IL-10 KO mice by non-parametric Mann-Whitney U test. (**B**) Cytokine production from BMDMs obtained from WT, OPN KO, IL-10 KO, and OPN/IL-10 DKO mice after stimulation with Pam3CSK4 (50 to 500 ng/ml), LPS (100 ng/ml), or ODN1585 (500 nM). N = 6 for each group. Error bars represent SEM. *P<0.05, one-way ANOVA with Bonferroni’s correction. (**C**) Gene expression of *NOS2*, *TNF-α*, *and IL-12p40* in LPMCs isolated from IL-10 KO and OPN/IL-10 DKO mice at 4, 8, and 12 weeks of age. N = 4 for each group. Gene expression of each target molecule was normalized by *GAPDH*. Error bars represent SD. *P<0.05 compared with IL-10 KO mice by non-parametric Mann-Whitney U test. (**D**) Changes of fluorescence intensity in TEPMs from WT, OPN KO, IL-10 KO, and OPN/IL-10 DKO mice after challenged with fluorescent particle conjugated-*E*.*coli*. Fluorescence intensity was measured at the indicated times and is shown in arbitrary units (AU). N = 6 for each group. Error bars represent SEM. *P<0.05 compared with TEPMs from WT mice; ✝*P*<0.05 compared with IL-10 KO mice by one-way ANOVA with Bonferroni’s correction. (**E**) Changes of fluorescence intensity in TEPMs from OPN KO mice after challenged with fluorescence particle conjugated-*E*.*coli* with or without recombinant OPN (rOPN) at 1, 10, 100, and 1000 ng/ml. Fluorescence intensity was measured at the indicated times and is shown in arbitrary units (AU). N = 6 for each group. Error bars represent SEM. *P<0.05 compared to TEPMs from OPN KO mice without rOPN (0 ng/ml) by one-way ANOVA with Bonferroni’s correction.

We further investigated the role of OPN in the innate immune responses by examining the effect of OPN on macrophage migration and functions, such as cytokine production and phagocytosis. First, we evaluated macrophage migration by quantifying *CD11b* gene expression in the colon. Gene expression of *CD11b* in the rectal tissues was similar between OPN/IL-10 DKO mice and IL-10 KO mice at 4 weeks of age, but it was significantly lower in IL-10 KO mice at 12 weeks of age (Fig 4A). Second, we generated BMDMs from WT, OPN KO, IL-10 KO, and OPN/IL-10 DKO mice, and stimulated them with TLR ligands. BMDMs from IL-10 KO and OPN/IL-10 DKO mice secreted higher amounts of TNF-α, IL-12p40, and IL-6 than BMDMs from WT and OPN KO mice after stimulation with Pam3CSK4 and LPS. The secreted amounts of these pro-inflammatory cytokines, however, did not differ significantly between WT and OPN KO mice, and IL-10 KO and OPN/IL-10 DKO mice after stimulation with any ligand (Fig 4B). Third, we isolated LPMCs from colonic tissues of IL-10 KO and OPN/IL-10 DKO mice, and examined their phenotype during colitis (Fig 4C). Gene expression of *NOS2* in LPMCs from OPN/IL-10 DKO mice was significantly lower at 4 weeks of age, but significantly higher at 8 and 12 weeks of age than that in LPMCs from IL-10 KO mice. LPMCs from OPN/IL-10 DKO mice expressed higher levels of *TNF-α* and *IL-12p40* than LPMCs from IL-10 KO mice at 4, 8, and 12 weeks of age.

Finally, we examined the effect of OPN on macrophage phagocytic function. The fluorescence intensity of TEPMs was decreased at each time point after challenge with fluorescence particle-conjugated *E*. *coli* in OPN KO and OPN/IL-10 DKO mice that lack iOPN compared to WT and IL-10 KO mice, respectively (Fig 4D). In addition, we evaluated the effect of exogenous OPN on macrophage phagocytic function by administering rOPN to TEPMs from OPN KO mice. Administration of rOPN at concentrations of 1, 10, and 100, but not 1000 ng/ml, increased the fluorescence intensity in TEPMs after challenge with fluorescent particle-conjugated *E*. *coli* (Fig 4E).

### Bacterial species increased in the mesenteric lymph nodes of OPN/IL-10 DKO mice compared to IL-10 KO mice

The paradoxically unenhanced uptake of *E*. *coli* in macrophages administered 1000 ng/ml rOPN implied that excessive rOPN might prevent the interaction between *E*. *coli* and macrophages. To confirm this phenomenon *in vivo*, we examined the microbiota composition in the MLNs of IL-10 KO and OPN/IL-10 DKO mice at 3 weeks of age. Multiple bacterial species were identified in the MLNs of OPN/IL-10 DKO mice compared to IL-10 KO mice (Table 2).

**Table 2:** Identified bacterial species in the mesenteric lymph nodes of IL-10 KO and OPN/IL-10 DKO mice.

Mouse	Identified bacteria
IL-10 KO	Propionibacterium acnes isolate Asn14
OPN/IL-10 DKO	Streptococcus sp. Smarlab 3301444
	Staphylococcus sp. CHNDP23
	Staphylococcus lentus
	Corynebacterium imitans
	Corynebacterium accolens
	Corynebacterium sp. CIP102857
	Staphylococcus sp. CNJ924 PL04

Bacterial species identified in the mesenteric lymph nodes of IL-10 KO and OPN/IL-10 DKO mice at 3 weeks of age.

N = 8 for each group.

## Discussion

The findings of the present study showed that disruption of OPN accelerated the onset of spontaneous colitis in a murine model of IBD. OPN synthesis was localized to colonic epithelia, and rapidly increased in response to inflammatory signals. OPN deficiency affected enteric microbiota *in vivo*, and both sOPN and iOPN regulated macrophage phagocytic function *in vitro*. Our findings suggest that OPN plays an important role in the onset of spontaneous colitis, affecting gut microbiota and macrophage phagocytic activity.

OPN KO mice did not develop spontaneous colitis. In the present study, however, OPN/IL-10 DKO mice had accelerated colitis compared to IL-10 KO mice, suggesting that OPN deficiency is a detrimental factor in the onset of spontaneous colitis.

The main cell type expressing OPN in the colonic tissue of IL-10 KO mice in response to inflammatory signals was then determined. Previous immunohistochemical analysis demonstrated increased OPN expression in the intestinal epithelial cells and infiltrating immune cells, such as macrophages and plasma cells, in the inflamed intestinal tissues of patients with IBD [14, 15]. Therefore, in the present study, we performed FISH analysis of OPN to identify the type of cell that synthesizes OPN in the inflamed intestinal tissues. FISH analysis revealed remarkably increased OPN synthesis in the colonic epithelia of IL-10 KO mice, even at 4 weeks of age, compared to WT mice, suggesting that epithelial cells are the main cells that synthesize OPN in the colonic tissues in response to inflammatory signals.

To elucidate the role of OPN secreted from colonic epithelia in mucosal immune responses, we examined the impact of OPN deficiency on enteric microbiota by comparing the enteric bacterial composition between IL-10 KO and OPN/IL-10 DKO mice at 3 weeks of age, just before the initiation of colitis. Surprisingly, T-RFLP analysis revealed a significant difference in the bacterial profiles with decreased abundance of *Clostridium* subcluster XIVa and increased abundance of *Clostridium* cluster XVIII in OPN/IL-10 DKO mice compared to IL-10 KO mice. Although the role of *Clostridium* cluster XVIII in mucosal immunity remains uncertain, *Clostridium* subcluster XIVa has a protective effect on DSS- and oxazolone-induced murine colitis by inducing regulatory T lymphocytes in the colon [30]. Thus, an intriguing notion is that OPN secreted from colonic epithelia prevents the development of colitis in IL-10 KO mice by affecting enteric bacteria, including *Clostridium* subcluster XIVa. Further study is necessary to elucidate how OPN affects enteric bacteria.

Next, we investigated the effect of OPN on macrophages, which are deeply involved in the initiation of colitis in IL-10 KO mice by sensing luminal bacterial components and producing a variety of pro-inflammatory cytokines [21]. We first examined *CD11b* mRNA expression levels in the colonic tissues of IL-10 KO and OPN/IL-10 DKO mice, which probably reflect macrophage numbers. There are several reports suggesting that OPN induces macrophage migration *in vitro* [4, 7]. The role of OPN in macrophage recruitment *in vivo*, however, still lacks consensus [9, 31]. In this study, *CD11b* mRNA expression in the rectal tissues of OPN/IL-10 DKO mice was similar to that in IL-10 KO mice at 4 weeks of age, but lower than that in IL-10 KO mice at 8 and 12 weeks of age. This finding suggests that the effect of OPN on macrophage recruitment depends on the phase of colitis in this model and might not contribute to the accelerated colitis in OPN/IL-10 DKO mice at 4 weeks of age. Then, we focused on the effect of OPN on cytokine production from macrophages. In previous studies, exogenous OPN enhanced the expression of several pro-inflammatory cytokines, such as IL-1β, TNF-α, IL-12, and IL-6, whereas it suppressed the expression of IL-10 in murine macrophages and human peripheral monocytes [8, 14, 32, 33]. Thus, theoretically, the lack of sOPN might lead to the amelioration of colitis in IL-10 KO mice by decreasing the production of pro-inflammatory cytokines from intestinal macrophages. In this regard, the rapidly deteriorating colitis in OPN/IL-10 DKO mice cannot be completely explained by the effect of sOPN deficiency on macrophage cytokine production. Therefore, we focused on the effect of iOPN on macrophage cytokine production. We stimulated BMDMs from WT, OPN KO, IL-10 KO, and OPN/IL-10 DKO mice with the ligands of TLR2, TLR4, and TLR9, all of which recognize bacterial components on the surface or inside of macrophages [34]. Comparison of the amounts of secreted pro-inflammatory cytokines, however, revealed no effect of iOPN deficiency on macrophage cytokine production. Taken together, these findings indicate that the effect of OPN deficiency on macrophage cytokine production does not contribute to the rapidly deteriorating colitis in OPN/IL-10 KO mice.

The macrophage phenotype during colitis was compared between IL-10 KO and OPN/IL-10 DKO mice to determine the effect of OPN on macrophage function *in vivo*. LPMCs isolated from OPN/IL-10 DKO mice expressed higher levels of TNF-α and IL-12p40 than those from IL-10 KO mice at 4 weeks of age, suggesting that intestinal macrophages are highly activated in OPN/IL-10 DKO mice at an early stage of colitis compared to IL-10 KO mice. On the other hand, gene expression of *NOS2*, a representative marker of pro-inflammatory macrophages [35], was significantly lower in LPMCs from OPN/IL-10 DKO mice at 4 weeks of age compared to IL-10 KO mice, whereas it became significantly higher after 8 weeks of age (Fig 4C). Several reports showed that OPN suppressed *NOS2* mRNA expression in murine macrophages *in vitro*, but the effect of OPN on *NOS2* mRNA expression and NO production in macrophages remains uncertain *in vivo* [36, 37]. Remarkably attenuated NOS expression was reported in the intestinal macrophages of OPN KO mice compared to WT mice in the DSS-induced colitis model [11]. Our findings suggest that the effect of OPN on *NOS2* expression in macrophages might be context-specific and dependent on the phase of colitis.

We then examined the effect of OPN on macrophage phagocytic function. We first assessed the effect of iOPN deficiency on macrophage phagocytic function. Shimizu et al. reported that BMDMs from OPN KO mice have impaired ability to phagocytose fluorescent beads [38]. Also, Heilmann et al. reported that macrophages isolated from the lamina propria of OPN KO mice with DSS-induced colitis have impaired ability to phagocytose fluorescein isothiocyanate-labeled *E*. *coli* [11]. Here, we showed impaired ability to phagocytose fluorescence-conjugated *E*. *coli* in TEPMs from OPN KO and OPN/IL-10 DKO mice compared to TEPMs from WT and IL-10 KO mice, respectively. Consistent with previous studies, our data clearly demonstrate that iOPN deficiency impaired the phagocytic function of macrophages, although the mechanism is not yet clear. We next assessed the effect of sOPN on the phagocytic function of macrophages using TEPMs from OPN KO mice. Administration of 1, 10, and 100, but not 1000 ng/ml rOPN enhanced the phagocytic function of TEPMs from OPN KO mice. Several reports have demonstrated that OPN can function as an opsonin to facilitate macrophage phagocytosis [39, 40]. Schack et al. showed enhanced phagocytosis of OPN-coated *Staphylococcus aureus* through α_x_β_2_ integrin expressed on the surface of phorbol myristate acetate-stimulated U937 cells [39]. Although we did not demonstrate the binding of rOPN to *E*. *coli* in this study, our findings suggest that rOPN enhances bacterial uptake in macrophages by binding to several bacterial species, including *E*. *coli*. In contrast, 1000 ng/ml rOPN did not enhance phagocytosis in TEPMs from OPN KO mice. This finding suggests that excessive rOPN between macrophages and bacteria might prevent their contact and thereby decrease bacterial uptake by macrophages. This phenomenon is consistent with the report by Heilmann et al. that a low dose (100 ng/ml), but not a high dose (500 ng/ml), of OPN enhances the ability of human macrophages to phagocytose fluorescein isothiocyanate-labeled *E*. *coli* [11]. In this regard, a large amount of OPN secreted from colonic epithelia in response to inflammatory signals might enhance mucosal defense by limiting the interaction between enteric bacteria and underlying mucosal immune cells, including macrophages, *in vivo*. The multiple bacterial species detected in the MLNs of OPN/IL-10 DKO mice compared to IL-10 KO mice at 3 weeks of age supports the notion of impaired mucosal defense in OPN/IL-10 DKO mice.

The findings of the present study suggest an important role of OPN in the onset of spontaneous colitis in mice. OPN deficiency affected the enteric microbiota, and both sOPN and iOPN regulated macrophage phagocytic function *in vitro*. Further studies regarding the direct effect of OPN on enteric bacteria, and the effect of OPN on macrophage phagocytic activity *in vivo* during spontaneous colitis will provide new insights into the role of OPN in the development of colitis as well as in the pathophysiology of IBD.
